# Mycobacterium marinum Infection and Aquatic Exposure: A Clinical Reasoning Pathway

**DOI:** 10.7759/cureus.84900

**Published:** 2025-05-27

**Authors:** Reem Sarsour, Monica Guirgus, Richard C Rice, Matthew Cappiello, Chitra Damodaran

**Affiliations:** 1 School of Medicine, California University of Science and Medicine, Colton, USA; 2 Orthopedics, Loma Linda University Medical Center, Loma Linda, USA; 3 Infectious Diseases, Loma Linda University Medical Center, Loma Linda, USA; 4 Infectious Disease, Veterans Affairs Medical Center, Loma Linda, USA

**Keywords:** acid-fast bacillus, aquatic infections, freshwater exposure, mycobacterium marinum, nontuberculous mycobacteria (ntm), orthopedic infections, tropical medicine

## Abstract

*Mycobacterium marinum* is a freshwater nontuberculous mycobacterial infection that can lead to cutaneous soft tissue infections in humans, with diagnosis frequently delayed if key historical details remain overlooked. We describe the case of a 57-year-old diabetic man who first presented with a left middle finger skin lesion, as well as a nodular area of erythema and swelling on his left ulnar styloid process that failed a trial of oral clindamycin. After multiple weeks of discoloration and progression of the ulnar lesion, he presented to an orthopedic clinic with purulent discharge from both affected areas, resulting in incision and drainage of small abscesses near his ulnar styloid as well as his left third metacarpophalangeal joint. After a thorough history taken by the infectious diseases consultant, it was revealed to be antecedent aquatic exposure; a punch biopsy for acid-fast bacilli grew *Mycobacterium marinum *on culture. Clinical resolution ultimately occurred after a four-month course of clarithromycin and ethambutol, a common regimen used in *Mycobacterium marinum *species with wild-type antibiotic sensitivity.

This case is representative of the clinical challenges involved in non-tuberculous mycobacteria diagnosis. Relevant epidemiologic risk factors included aquatic exposure, while medical risk factors included poorly controlled diabetes. The exam was characteristic of common disease presentations, as the patient presented with multiple cutaneous lesions in a sporotrichoid distribution. Differential diagnosis can include bacterial infections, as well as multiple endemic dimorphic fungal and parasitic infections. A clinical reasoning pathway is proposed to streamline the diagnosis of *Mycobacterium marinum and* ensure early and accurate identification.

## Introduction

*Mycobacterium marinum* (*M. marinum*) is a freshwater, gram-positive, acid-fast bacillus classified as a slow-growing non-tuberculous mycobacteria. It is associated with the development of a cutaneous soft tissue infection, most commonly affecting the hands and distal upper extremities. In the United States, *M. marinum *is a rare infection, leading to only 2.7 cases per 100,000 individuals annually [1]. Also known as 'swimming pool granuloma' due to its original discovery in a contaminated pool in Sweden, infection commonly occurs secondary to domestic pools as well as aquaria and other marine environments [2]. Lesions caused by *M. marinum* can resemble other cutaneous and lymphatic infections, as well as non-infectious skin conditions [3]. 

The diagnosis of *M. marinum* is often delayed due to overlooking key historical details such as previous marine exposures. It is a photochromogen producing yellow pigment on light exposure, with preferential growth on Lowenstein Jensen media at 32°C, in contrast to *Mycobacterium tuberculosis*, which grows more optimally at 37°C. Immunocompromised individuals are at increased risk for severe and/or disseminated infection [1], and treatment is complicated due to the involvement of a large number of strains that often have multidrug resistance [4]. The purpose of this case report is to contribute a piloted diagnostic pathway to appropriately suspect *M. marinum* versus similar skin diagnoses, by correlating the patient's clinical presentation with established patterns in existing *M. marinum* literature.

## Case presentation

A 57-year-old male with a past medical history significant for brittle diabetes mellitus type 2 (DM2) presented with a skin lesion at the distal tip of the left middle finger at a diabetic fingerstick site. At the time, his hemoglobin A1C was 10 (Table 1), reflecting poorly controlled diabetes and a functionally immunocompromised state. Over two weeks, the patient noticed that the lesion progressively expanded into a nodular area of erythema and swelling at the left ulnar styloid process. He initially monitored his lesions at home, and eventually scheduled a primary care visit after four weeks of symptoms. At this visit, the patient was empirically prescribed clindamycin 150 mg four times daily for 10 days. The next day after his visit, the patient visited the emergency department due to continually worsening swelling, erythema, and purple discoloration in the left hand. The emergency department increased his dose to 300 mg three times daily and referred him for an orthopedic surgery consult through his contracted health insurance program for military veterans. 

**Table 1:** Laboratory Values on Initial Visit CO2: Carbon dioxide, eGFR: Estimated glomerular filtration rate, ALP: Alkaline phosphatase, ALT: Alanine aminotransferase, AST: Aspartate aminotransferase

Parameters	Value	Units	Normal range
Sodium	140	mMol/L	136-144
Potassium	4.4	mMol/L	3.6-5.1
Chloride	107	mMol/L	101-111
CO2	26	mMol/L	22-32
Urea Nitrogen	23	mg/dL	8-20
Creatinine	1.5	mg/dL	.64-1.27
eGFR	54.3	mL/min	>=60
Glucose	69	mg/dL	74-118
Calcium	9.2	mg/dL	8.9-10.3
Albumin	3.8	mg/dL	3.5-4.8
Total bilirubin	0.5	mg/dL	0.2-1.2
ALP	71	IU/L	32-91
ALT	18	IU/L	17-63
AST	16	IU/L	15-41
Hemoglobin A1C	10		4.6-6.0
White blood cells	8.11	billion/L	4-10
Hemoglobin	14.7	trillion/L	13.5-17.5
Hematocrit	44.8	%	40-53
Platelets	199	billion/L	150-450

The patient ultimately attended his orthopedic surgery consultation after two months of symptoms. The lesion had continued to worsen in swelling, with palpable fluctuance and violaceous cutaneous discoloration. A central ulcer was noted on the dorsum of the left hand. Additionally, physical exam suggested abscess formation, with purulent discharge present near the left hand third metacarpophalangeal joint as well as the left ulnar styloid process. Three X-ray views were taken of the left hand to assess for osteomyelitis; radiographs showed only mild atherosclerotic changes in the hand. The patient underwent irrigation and debridement in the orthopedic surgery clinic. Clindamycin was extended for a total of 14 days, and double-strength trimethoprim/sulfamethoxazole was added twice a day for 14 days.

**Figure 1:**
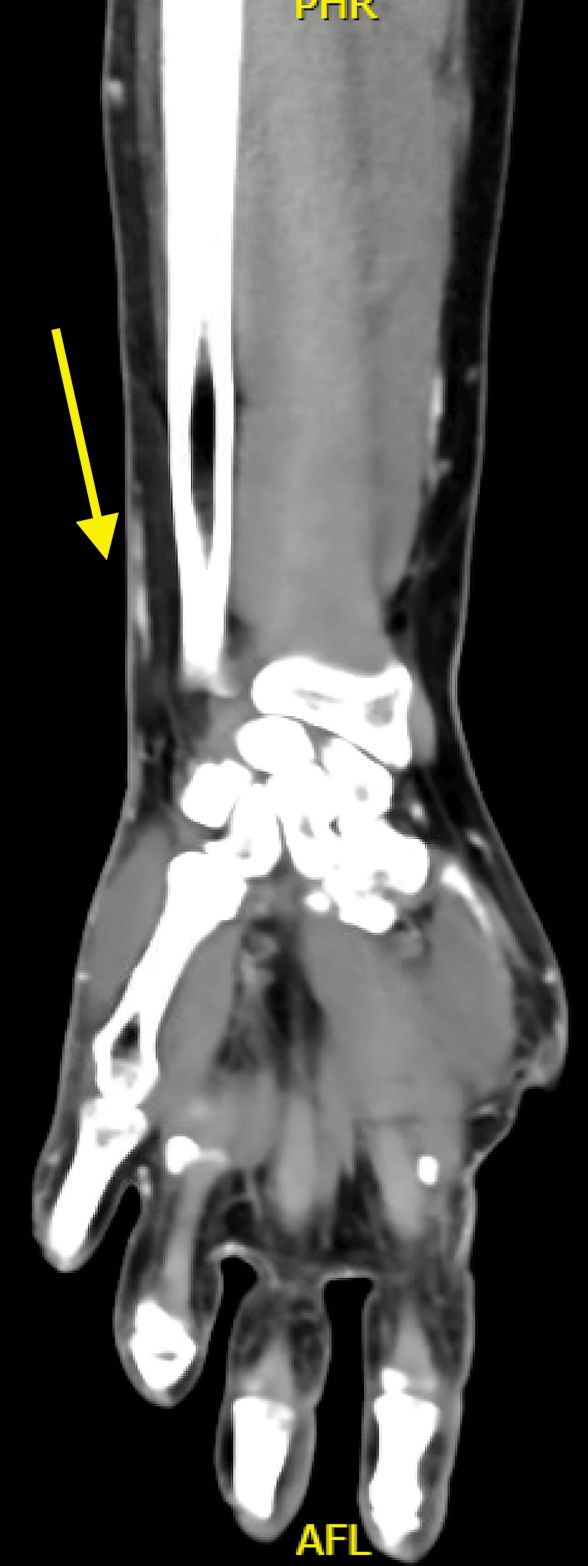
CT scan of upper extremity, coronal view The arrow points to fat stranding, which is concerning for soft tissue infection. AFL: Anterior forearm left-sided

**Figure 2:**
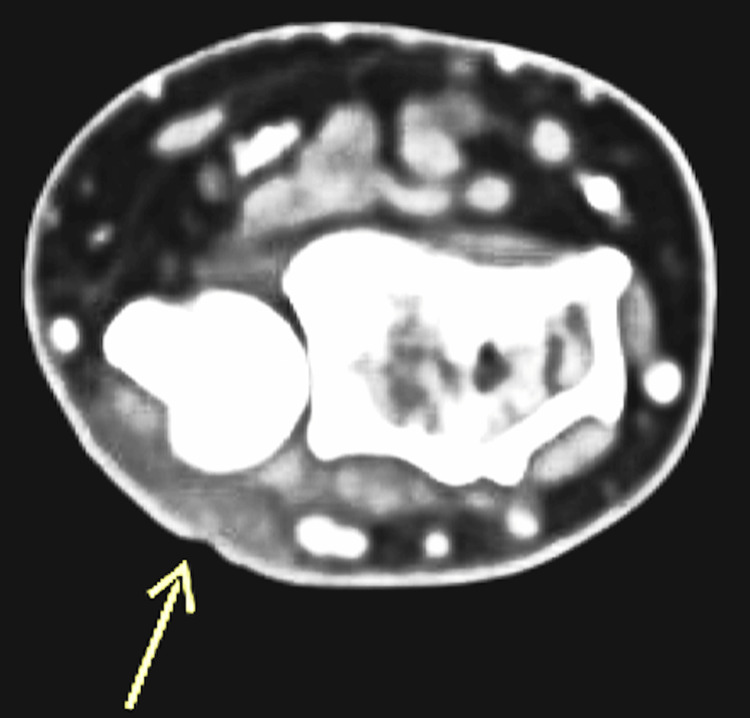
CT scan of upper extremity, sagittal view The arrow points to the fat stranding, which is concerning for soft tissue infection without osteomyelitis.

The patient returned to the clinic for the two-week follow-up. Minimal improvement was seen, despite completing his antibiotic course. The infectious diseases team was consulted for a more thorough exposure history. Notably, the patient reported that he picked leaves out of his unchlorinated pool filled with green algae. In addition, he cleaned the rocks in his fish tank by sticking his bare hand into the tank to remove the rocks. These freshwater exposures increased the infectious diseases consultant's suspicion of mycobacterial infection. A CT scan was obtained (Figures 1-2), showing subcutaneous fat stranding and edema along the dorsal-ulnar aspect of the wrist with adjacent mild skin irregularity, otherwise negative for abscess, soft tissue gas, or osteomyelitis. The patient's course of the above antibiotics was extended, and the infectious diseases team also recommended a punch biopsy from the left hand to exclude mycobacterial and fungal disease. 

**Figure 3:**
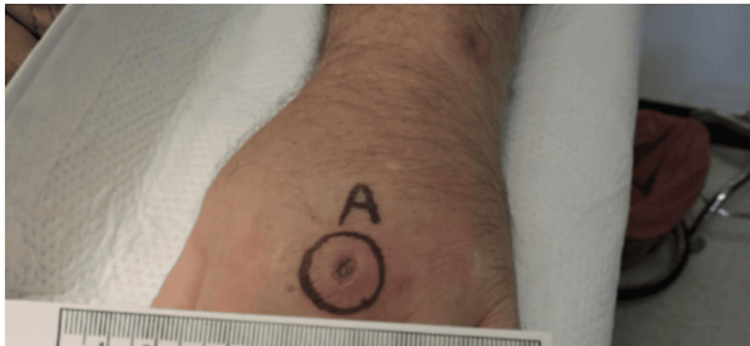
Punch biopsy of lesion taken from the center of the dorsum of the left hand

**Figure 4:**
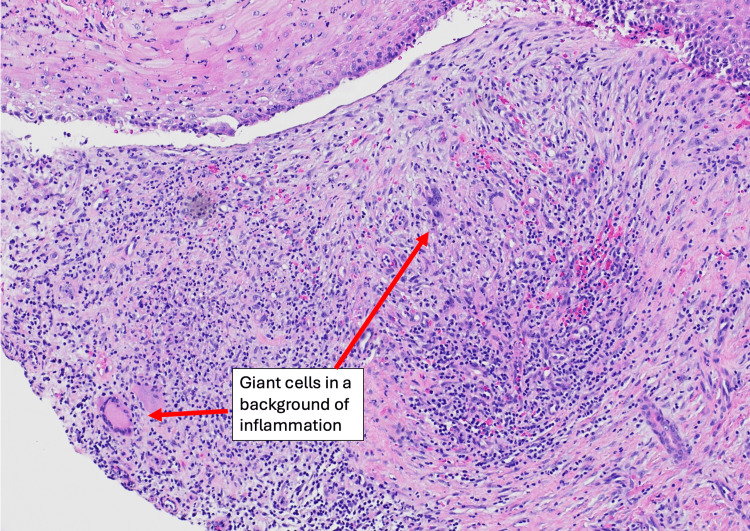
Tissue histopathology of skin biopsy specimen Granulomatous inflammation with giant cells, lymphocytes, and macrophages are visible.

**Figure 5:**
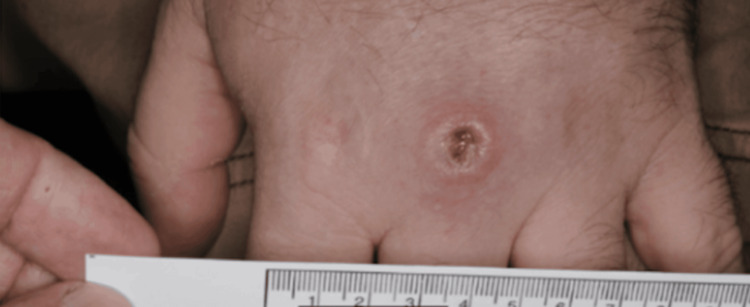
Close-up of the lesion on the dorsum of left hand after the initial treatment with clarithromycin and ethambutol for 30 days

**Figure 6:**
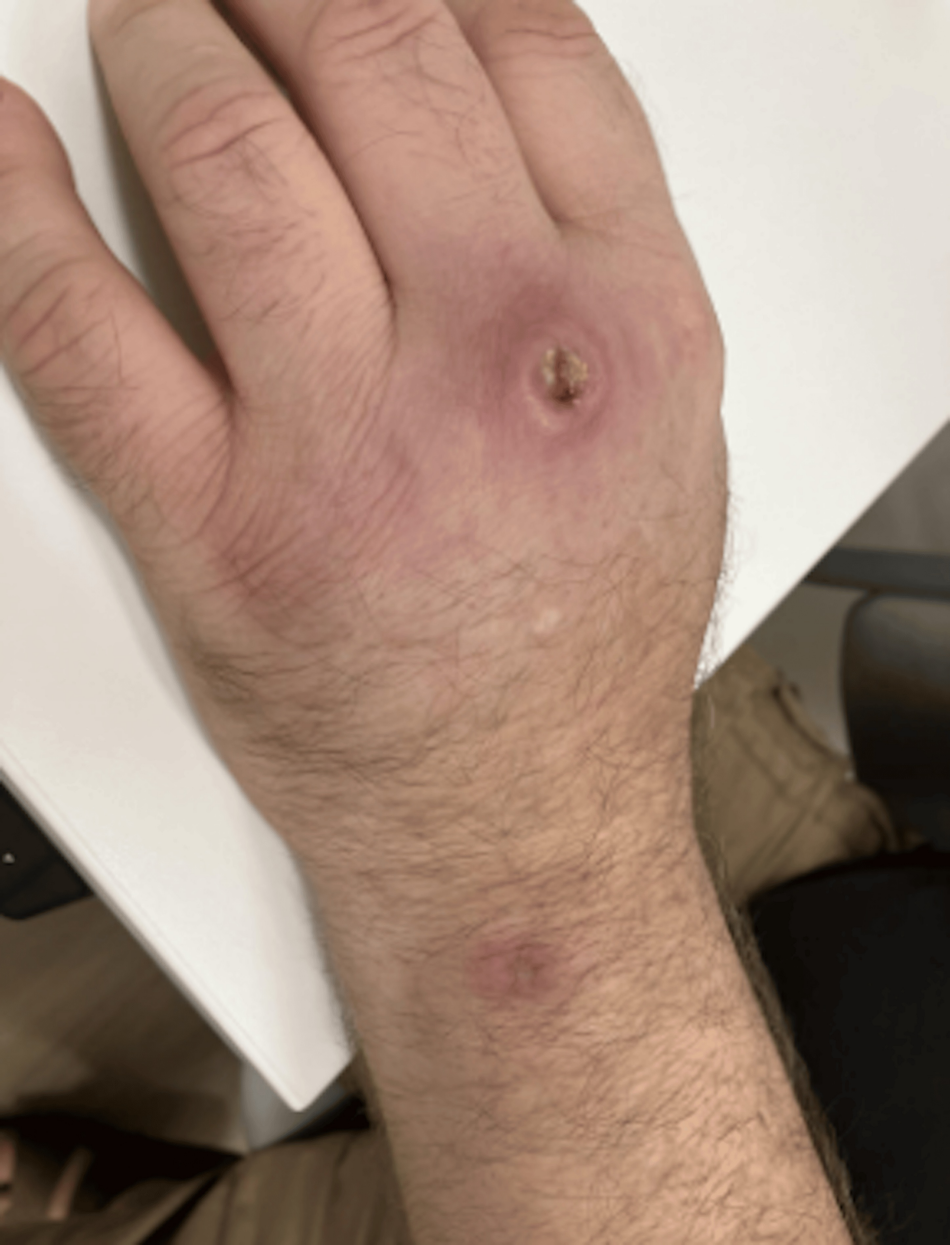
Exterior view of the lesion on the dorsum of left hand after initial treatment with clarithromycin and ethambutol for 30 days

**Figure 7:**
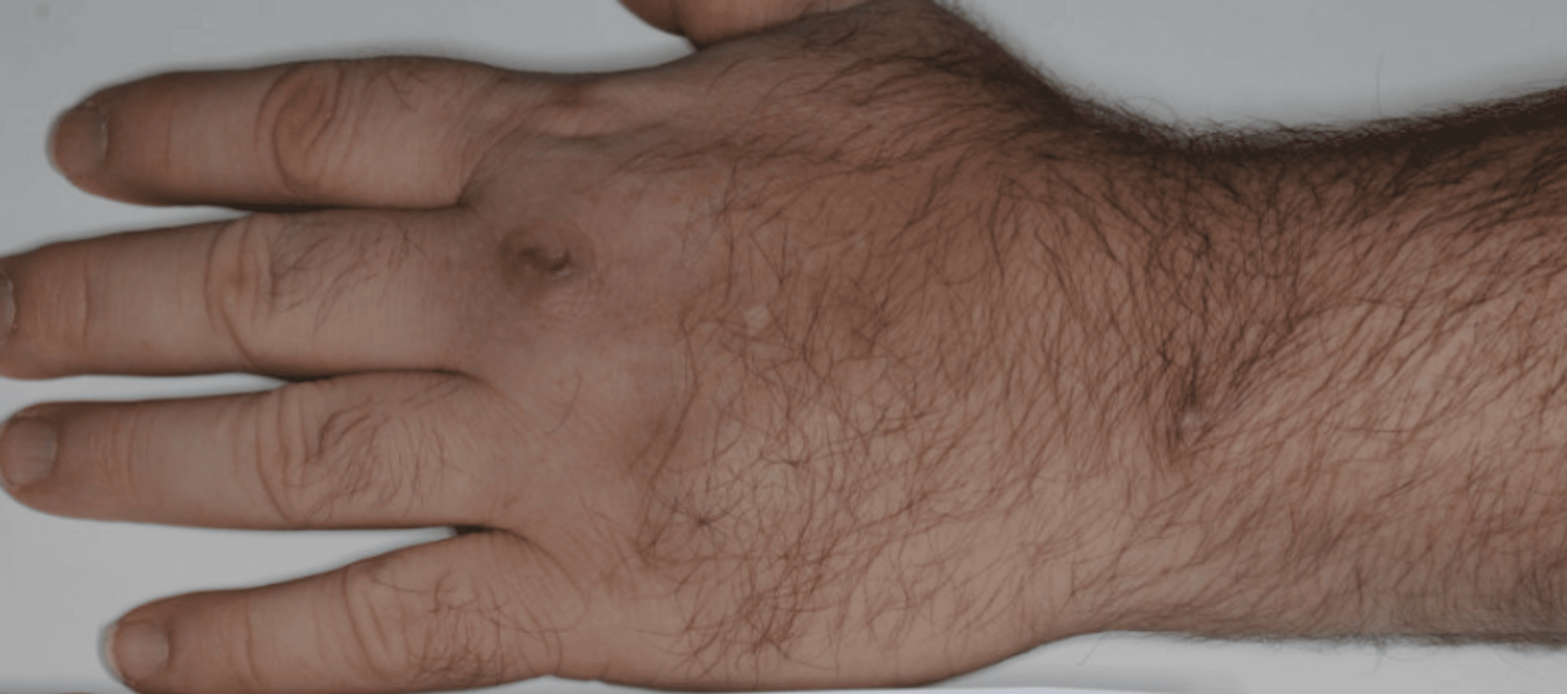
Healed lesion after four months of ethambutol and clarithromycin treatment

The patient underwent a punch biopsy of his left hand. This was cultured for 30 days on acid-fast bacilli (AFB) culture, as well as Gomori methenamine silver (GMS) stain, potassium hydroxide (KOH) preparation, and periodic acid-Schiff (PAS) stain (Figure 3). Tissue histopathology showed skin with granulomatous inflamed tissue (Figure 4). Cultures confirmed *M. marinum*, with susceptibility testing confirming that the isolate was sensitive to clarithromycin, rifampin, moxifloxacin, ciprofloxacin, amikacin, and linezolid. The patient was started on clarithromycin 500 mg twice daily and ethambutol 2400 mg daily. After four months of treatment, the patient’s lesions eventually resolved; interval improvement was seen on the 30-day follow-up (Figures 5-6) as well as the four-month follow-up (Figure 7). 

## Discussion

In this case report, identification of the etiology of the infection spanned a total of five months from initial assessment. Initial failed treatment for bacterial skin and soft tissue infection led to specialist consultation, with final diagnosis occurring on culture and tissue histopathology. A study investigating 46 *M. marinum* cases showed a median time to diagnosis of 3.6 months, with the range spanning from 2.3 to 6.1 months [5]. Delayed diagnosis is sometimes related to antecedent prolonged treatment for bacterial soft tissue infection, as well as providers overlooking epidemiologic risk factors, including marine exposures. Delayed diagnosis is also related to *M. marinum’s *variable clinical presentation, including papulonodular to ulcerating skin lesions with sporotrichoid pattern and other nonspecific symptoms, including painful swelling, stiffness, numbness, local pain, limitation of motion, or sinus tracts with discharge [6-8]. Diagnosis requires clinical suspicion, especially as gold-standard testing such as culture and polymerase chain reaction (PCR) requires several weeks for laboratory analysis [9]. Since clinical presentation and progression of disease are often non-specific, a wide differential diagnosis can also explain symptoms such as blastomycosis, histoplasmosis, cryptococcosis, nocardiosis, leishmaniasis, and other diseases caused by non-tuberculous mycobacteria (NTM) [10]. Additionally, non-infectious conditions such as skin tumors, foreign-body granulomatous reactions, and sarcoidosis could be considered.

Targeted diagnostics were necessary to confirm the diagnosis in this patient, including punch biopsy for histopathology as well as AFB culture and sensitivity testing. The patient was ultimately treated with clarithromycin and ethambutol as indicated by societal guidelines [11,12], and lesions showed immediate improvement within 30 days of treatment. Treatment may be complicated due to the involvement of a large number of strains that can display multidrug resistance [4]. Optimal treatment regimen in instances of suspected resistance would require antibiotic sensitivity testing; common antibiotics that demonstrate sensitivity include ethambutol, clarithromycin, and streptomycin [13]. However, since antibiotic sensitivity testing results require several weeks and *M. marinum *often responds to empiric first-line treatment with clarithromycin and ethambutol, empiric antibiotics with this patient’s regimen are traditionally continued, and improvement is monitored clinically [11]. Ultimately, drug susceptibility testing in our patient confirmed that the treatment regimen was appropriate. 

When comparing the case presented in this study to prior reports of *M. marinum,* key similarities were noted in demographics, comorbidities, and course of disease. *Mycobacterium marinum *seems to favor middle-aged adults. Our patient was 57 years old, and the reported average age of diagnosis of M. marinum is 43 years in the literature [14]. Furthermore, *M. marinum* infections tend to occur more frequently in men, with worse outcomes also noted in men [9]. In addition, our patient suffered from diabetes mellitus, which is prevalent among 27% of cases of *M. marinum* infection in some literature [15]. The presentation of our patient’s initial skin infection on the dorsum of the hand is consistent with the natural history of the disease, as 93% of *M. marinum* infections present initially on the hand in other literature [16]. Our patient reported a history of aquatic exposure, and 87% of patients with a definitive *M. marinum *diagnosis reported previous exposure to aquatic environments [17]. Infection of this pathogen most commonly occurs in the finger or hand from the entry of the bacteria through pre-existing wounds and may continue to spread within the lymphatic system. It localizes to the skin due to its optimal growth at 30°C, but can develop attenuated growth at 37°C, allowing for deeper skin infections and osteomyelitis. Although presentation may vary, infection commonly begins as a small red papule that enlarges over time into a violaceous nodular plaque presenting as a sporotrichoid nodular pattern on the skin. Without treatment, these infections may progress to the bone, leading to deep infections requiring orthopedic care, including tenosynovitis, osteomyelitis, arthritis, and bursitis. 

**Figure 8:**
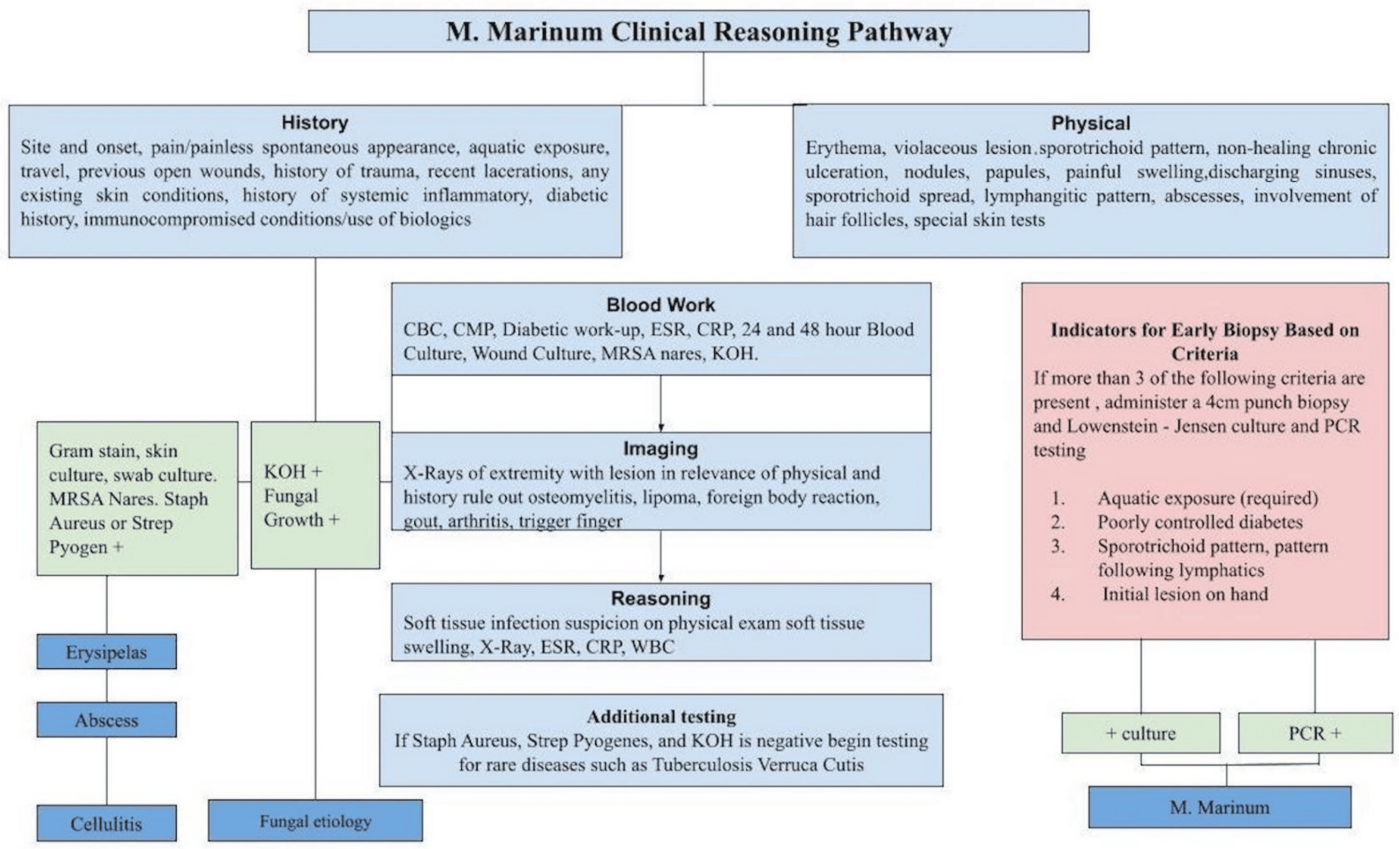
Clinical reasoning pathway for suspected M. marinum soft tissue infection Flowchart created by authors. *M. marinum*: *Mycobacterium marinum*, KOH: Potassium hydroxide, MRSA: Methicillin-resistant Staphylococcus aureus, CBC: Complete blood count,  CMP: Complete metabolic panel, ESR: Erythrocyte sedimentation rate, CRP: C-reactive protein, WBC: White blood count, PCR: Polymerase chain reaction

Due to the clinical challenges involved in *M. marinum* diagnosis, this study proposes a clinical reasoning pathway (Figure 8) to streamline diagnosis, ensuring early and accurate identification of the disease. To our knowledge, no previous studies in the literature have provided a clinical algorithm for a time-sensitive diagnosis of *M. marinum*. Review of patient demographics, comorbidities, exposures, and clinical presentation in the prior literature led to the construction of a clinical reasoning pathway to discern *M. marinum* from differential diagnoses such as cellulitis, abscess, fungal infections, and tuberculosis verruca cutis [17,18]. Our pathway suggests that the clinician can undertake diagnostic testing of* M. marinum* with early biopsy if greater than three specific criteria are met, namely freshwater or marine exposure to an open wound, history of diabetes mellitus, soft tissue infection presenting initially on the hand, and sporotrichoid pattern on exam.

A crucial aspect of skin and soft tissue infections involves a detailed patient history, including aquatics, recent travel, gardening, contact sports, shared living facilities, and medications that may increase susceptibility to infection, such as biologic and immunosuppressive therapy. It is also essential to inquire about systemic and chronic inflammatory conditions that contribute to the net state of immunosuppression in the patient, including diabetes mellitus, malignancy, and HIV. Autoimmune skin diseases, including psoriasis, should be noted. Physical examination should assess for signs of erythema, purple discoloration, healing, scars, nodules, ulceration, violaceous lesions, sporotrichoid pattern, and abscess. 

Following history and physical exam, standard tests should be ordered, including complete blood count, Westergren (erythrocyte sedimentation rate (ESR)), and C-reactive protein inflammatory markers, blood cultures, wound culture, and KOH preparation. Wound cultures that grow gram-positive cocci positive for *Staphylococcus aureus* or *Streptococcus pyogenes* may point towards cellulitis, erysipelas, or abscess. If the KOH fungal test is positive on skin scraping, it suggests fungal etiology. Radiographs can assess for osteomyelitis and can also confirm the presence of lipoma, foreign body reaction, gout, arthritis, or trigger finger. If soft tissue infection is suspected but cultures remain negative, one can consider additional culture testing, including AFB culture and smear. If at least three proposed criteria are met, and positive cultures additionally confirm *M. marinum* infection, it can guide appropriate treatment per societal guidelines [11,12]. Definitive diagnosis requires a positive tissue culture, with a mean detection time of 25 days and mycobacterial identification and antimicrobial susceptibility results available in 26 days [8]. Although no randomized controlled trials are available for treatment regimens, treatment usually takes months [1], with longer courses recommended in immunocompromised hosts. Chronic lifetime treatment is sometimes necessary for suppression. 

## Conclusions

This case report exemplifies the hurdles a medical team may face when diagnosing and treating a rare infectious disease such as* M. marinum*. Detection can be elusive and cause months of discomfort, along with the risk of more deep-seated infections such as osteomyelitis. In this case, identification of the etiology of infection spanned a total of six months from initial assessment and required a multidisciplinary team, including infectious diseases and orthopedic surgery. Epidemiologic history of fish tank exposure heightened the team’s suspicion for an aquatic source of disease. Targeted diagnostics were employed to confirm the diagnosis, including punch biopsy for histopathology as well as AFB culture and sensitivities. The patient was ultimately treated with clarithromycin and ethambutol as indicated by sensitivity testing and was considered fully healed approximately seven months after initial presentation. Our clinical reasoning pathway outlines a piloted systematic approach for diagnosing and managing infections caused by *M. marinum*, based on both this case presentation as well as history, exam, and lab criteria in similar case presentations. This pathway can help expedite the management of patients with similar symptoms.
